# Dr. Luke

**Published:** 1829-05

**Authors:** 


### DR. LUKE.

We have also to lament the death of this highly respectable physician, who died on Monday last; we believe of diseased heart.—*Ib.*

